# Quinolinic acid as trigger/biomarker of dysosmia/dysgeusia in patients with acute coronavirus disease 2019: A retrospective case-control study

**DOI:** 10.1016/j.bbih.2026.101175

**Published:** 2026-01-12

**Authors:** Jun Tsukiji, Shiro Koizume, Tomoko Takahashi, Shuji Murakami, Hiroyuki Takahashi, Sachiyo Mitsunaga, Sho Nakamura, Hiroto Narimatsu, Yohei Miyagi

**Affiliations:** a Department of Prevention and Infection Control, Kanagawa Cancer Center, Yokohama, 241-8515, Japan; b Molecular Pathology and Genetics Division, Kanagawa Cancer Center Research Institute, Yokohama, 241-8515, Japan; c Department of Thoracic Oncology, Kanagawa Cancer Center, Yokohama, 241-8515, Japan; d Department of Medical Oncology, Kanagawa Cancer Center, Yokohama, 241-8515, Japan; e Department of Oral and Maxillofacial Surgery, Kanagawa Cancer Center, Yokohama, 241-8515, Japan; f Cancer Prevention and Control Division, Kanagawa Cancer Center Research Institute, Yokohama, 241-8515, Japan; g Graduate School of Health Innovation, Kanagawa University of Human Services, Kawasaki, 210-0821, Japan

**Keywords:** COVID-19, Dysosmia, Dysgeusia, Indoleamine 2,3-dioxygenase (IDO), Kynurenine- tryptophan ratio (KTR), Quinolinic acid

## Abstract

**Background:**

Impaired smell/taste sensation (dysosmia/dysgeusia) are common manifestations of coronavirus disease 2019 (COVID-19). Scattered peripheral chemoreceptors and directly innervating central nerves from the brain to the receptors are responsible systems for perception in the human body. The shared neurotransmitter serotonin (5-HT) and neuroimmune modulators of the kynurenine (Kyn) pathway (KP) are metabolites derived from tryptophan (Trp). The synthesis of KP metabolites is initiated by the rate-limiting enzymes indoleamine 2,3-dioxygenase (IDO) and tryptophan 2,3-dioxygenase (TDO). Severe acute respiratory syndrome coronavirus 2 (SARS-CoV-2) can activate Trp metabolism. Therefore, we investigated whether serum metabolites of Trp and IDO/TDO activity could serve as biomarkers for assessing smell/taste impairment (dysosmia/dysgeusia) in patients during the acute phase of COVID-19.

**Methods:**

We conducted a retrospective case-control study. Among patients admitted with acute COVID-19 to our hospital between September 13, 2021, and September 30, 2023, those whose chief complaints included dysosmia/dysgeusia at admission were identified. These symptoms were confirmed by the attending physician for COVID-19. Patients were stratified based on the presence or absence of dysosmia and/or dysgeusia.

In both patient groups, serum concentrations of Trp, 5-HT, Kyn, kynurenic acid (KYNA), and quinolinic acid (QUIN) were measured using enzyme-linked immunosorbent assay. IDO/TDO activity was expressed as Kyn–Trp ratio (KTR). The relationships between these biomarkers and dysosmia/dysgeusia, as well as other clinical parameters and outcomes, were evaluated.

**Results:**

Of 520 patients admitted with COVID-19, 95 met the inclusion and exclusion criteria. Among them, 26 patients with dysosmia/dysgeusia (group A) and 26 patients without these symptoms (group B) were analyzed. No significant intergroup difference was observed in the average timepoint at blood sampling after COVID-19 onset (post-day from onset: pdo) (4.69 ± 2.51 days in group A *vs*. 3.62 ± 2.22 in group B). Group A showed significantly lower Trp levels [median 9.70 μg/mL (range 4.59–13.89) *vs*. 10.40 (7.52–13.34), *p* = 0.031], and higher KTR [61.34 (40.47–384.2) *vs*. 53.52 (26.13–86.64), *p* < 0.037] and QUIN levels [574.39 nM (100.39–11909) *vs*. 443.65 (83.09–998.3), *p* < 0.0169]. No significant differences were observed in 5-HT or KYNA levels between groups. Almost all cases of dysosmia involved anosmia/hyposmia and were significantly correlated with non-vaccination status with mRNA vaccine (*p* = 0.017). In contrast, dysgeusia exhibited heterogeneous manifestations, primarily ageusia or hypogeusia, followed by hypersensitivity to salty taste, and was not correlated with vaccination status.

**Conclusion:**

Clinically, serum KTR and QUIN levels may serve as useful biomarkers for assessing dysgeusia/dysomia during acute COVID-19. Furthermore, vaccination may play an important preventive role, particularly against dysosmia.

## Highlights

• Neurological signs with COVID-19 may be due to neuromediator imbalance.
• COVID-19 patients stratified by presence/absence of dysgeusia/dysosmia were tested.
• Serum Kyn-Trp ratio & QUIN levels are potent biomarkers of dysgeusia/dysosmia.
• Targeting kynurenine pathway is a promising strategy to manage dysosmia/dysgeusia.
• Vaccination could serve as an effective measure for preventing dysosmia.

## 1 Introduction

Caused by severe acute respiratory syndrome coronavirus 2 (SARS-CoV-2), coronavirus disease (COVID-19) was first declared a pandemic in 2020 and continues to exert great pressure on healthcare systems worldwide (Bajema et al., 2025). Following its sudden emergence, COVID-19 generated significant concern due to its potential for causing critical illness, including severe respiratory failure and thrombotic conditions and acute respiratory distress syndrome (ARDS). Notably, ARDS was associated with peculiar symptoms such as “silent hypoxemia (happy hypoxia),” characterized by the absence of dyspnea despite life-threatening hypoxemia (Brouqui et al., 2021; Couzin-Frankel, 2020). A vast proportion of patients continue to experience a myriad of long-lasting manifestations, such as ageusia (loss of taste); anosmia (loss of smell); and memory and attention deficits or neurocognitive impairment (brain fog); this presentation has been comprehensively defined as “long COVID” (Davis et al., 2023). However, the etiopathogenesis of these symptoms and their resolution remain unclear.

In mammals, including humans, peripheral tissue chemoreceptors are responsible for sensory functions, such as taste sensations mediated by taste buds (TB) (Barretto et al., 2015; Larson et al., 2015; Roper and Chaudhari, 2017) and olfaction through olfactory bulb glomeruli (OBG) and solitary chemoreceptor cells (SCC) (Brill et al., 2016; Abraham and Mathew, 2019). They also detect hypoxia/hypoxemia via pulmonary neuroepithelial bodies (NEB) and carotid bodies (CB), which perceive decreased partial oxygen pressure in the airway and blood, respectively (Youngson et al., 1993; Noguchi et al., 2020; Prabhakar and Peng, 2017). In spite of scattered distribution, these chemoreceptors have a functional resemblance to the endocrine paracrine system through shared neuromediators such as that in cells of the Amine Precursor Uptake and Decarboxylation (APUD) system (Abraham and Mathew, 2019; Youngson et al., 1993; Boyd, 2001). Chemoreceptor cells utilize a shared bioactive monoamine neurotransmitter, serotonin (5-hydroxytryptamine [5-HT]) (Roper and Chaudhari, 2017; Brill et al., 2016; Youngson et al., 1993; Gu et al., 2014). Enterochromaffin cells (ECs) in the gut, which function as chemoreceptors (Kidd et al., 2008; Bellono et al., 2017), are the primary source of 5-HT in the human body (Linan-Rico et al., 2016). Notably, clustered receptors, such as TB, CB, and NEB, exhibit highly similar morphological structures (Youngson et al., 1993). Additionally, both cluster-type and single-cell receptors (SCC and EC) establish specialized direct connections with the central nervous system (CNS) via nerves, including the olfactory (OBG), trigeminal (SCC), glossopharyngeal (TB and CB), and vagus nerves (TB, NEB, SCC, and EC) (Abraham and Mathew, 2019; Caretta and Mucignat-Caretta, 2022). In the gustatory system, sensory neurons in TB are specialized to detect compounds that elicit sweet, salty, sour, bitter, and umami (savory) tastes (Barretto et al., 2015). Moreover, certain chemoreceptors (TB, CB, and OBG) contain glia-like cells with roles analogous to those of CNS glial cells, such as astrocytes and microglia (tissue-resident macrophages in the CNS), which perform essential functions (Roper and Chaudhari, 2017; Sobrino et al., 2020; Pardal et al., 2007; Oprych et al., 2017). These glia/glia-like cells are preferentially targeted by SARS-CoV-2 (Andrews et al., 2022; Kase et al., 2023; Brann et al., 2020). Given the potential for direct SARS-CoV-2-induced dysfunction in these cells, understanding the role of neurotransmitters and neuromodulators in glia/glia-like cells and the sensory systems linking peripheral chemoreceptors to the brain is crucial for elucidating the etiology of COVID-19-associated neuropathies.

5-HT plays vital physiological roles in the immune, vascular, and digestive systems, as well as in the CNS (Herr et al., 2017). It is synthesized from L-tryptophan (Trp), an essential amino acid. Trp metabolites in the kynurenine (Kyn) pathway (KP), distinct from the 5-HT pathway, function as key neuro/immunomodulators and signaling molecules alongside 5-HT, playing a critical role in autoimmune and neurodegenerative diseases (Platten et al., 2019; Vécsei et al., 2013). Notably, the synthesis of KP metabolites is regulated by glial cells and macrophages (Platten et al., 2019; Krause et al., 2011; Stone et al., 2022). Both Trp metabolites are associated with COVID-19 severity, with serum 5-HT levels decreasing as KP metabolite concentrations fluctuate (i.e., Trp levels decline while Kyn levels rise) (Saito et al., 2022; Thomas et al., 2020; Shen et al., 2020). A recent study suggested that 5-HT depletion, linked to vagus nerve dysfunction, is a major contributor to long COVID (Wong et al., 2023). Thus, the neurological symptoms of COVID-19 may be closely associated with the dysregulation of Trp and its metabolites.

Although symptom prevalence decreases over time following acute illness, many patients continue to experience persistent COVID-19 symptoms long after the acute phase has resolved (Montoy et al., 2023). Taken together, these findings support the hypothesis that the diverse neurological manifestations observed in patients with long COVID may be associated with prolonged dysregulation of neurotransmitters and modulators, particularly abnormal modulation of shared neuromediators such as 5-HT or KP metabolites, beginning early after COVID-19 onset. Moreover, although the emergence of mRNA vaccination against SARS-CoV-2 has greatly contributed to preventing aggravation during acute COVID-19, its potential suppressive effects on dysosmia/dysgeusia warrant further investigation.

In this study, we aimed to investigate the serum concentrations of Trp and its metabolites (5-HT and KP metabolites) in samples collected at an early timepoint (<5 days) following COVID-19 onset in patients stratified by the presence or absence of dysgeusia/dysosmia. Additionally, we evaluated whether correlations exist between the concentrations of these compounds and the clinical manifestations of COVID-19.

## 2 Materials and methods

### 2.1 Study location and participants

**Fig. 1:**
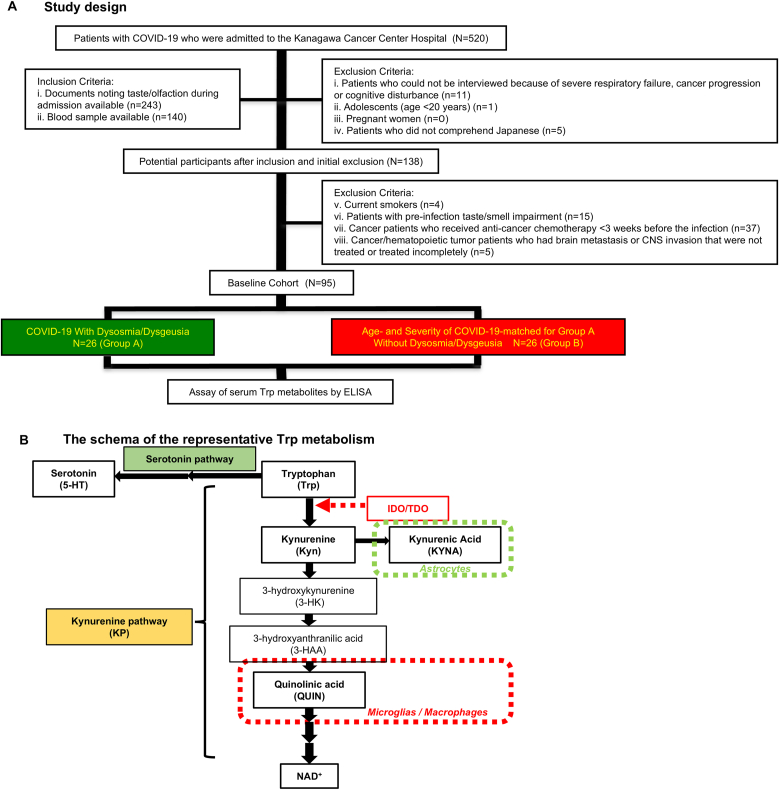
Study design, a summary of enrolled patients with COVID-19, and the schema of the representative Trp metabolism. (a) A case-control study of 520 patients with COVID-19 who had been admitted to our hospital had received chest HRCT examinations. After initial exclusion, inclusion, and second exclusion, 95 patients were selected as potential participants for the baseline study. After 26 patients with COVID-19 with dysosmia and/or dysgeusia were recruited from this baseline as a case subgroup (group A), an age-, disease severity- and patients with cancer-matched control subgroup without dysosmia/dysgeusia was selected from the baseline cohort (group B). (b) The schema reveals the representative pathway of Trp metabolism and type of glia cells producing the distinctive metabolite. COVID-19, coronavirus disease 2019; HRCT, high-resolution computed tomography; IDO, indoleamine 2,3-dioxygenase; NAD, nicotinamide adenine dinucleotide; TDO, tryptophan 2,3-dioxygenase; Trp, tryptophan.

This single-center retrospective case-control study was conducted at Kanagawa Cancer Center Hospital, Yokohama, Japan. The participants were patients with COVID-19 who were referred by the Kanagawa prefectural government or hospital medical staff, as well as regular patients with cancer treated at the hospital (Fig. 1a and Table 1). This study included inpatients hospitalized with confirmed COVID-19 between September 13, 2021, and September 30, 2023. As this study included only patients admitted to our hospital, healthy individuals could not be enrolled. Data collected included age, sex, COVID-19 severity, laboratory data, comorbidities, and treatment (Table 2, Supplementary Tables S1 and S2).

**Table 1:** Summary of patients with COVID-19 with and without dysosmia/dysgeusia.

Group	Patient ID (age, sex)	pdo (days)	Severity of COVID-19	Dysgeusia	Dysosmia
**A**	A-1 (41, F)	5	moderate grade I	no complaint	anosmia
	A-2 (51, F)	5	mild	no complaint	hyposmia
	A-3 (53, M)	6	moderate grade I	hyper-sensation for salty	anosmia
	A-4 (51, M)	9	moderate grade II	hypogeusia	no complaint
	A-5 (50, M)	4	moderate grade I	hyper-sensation for salty	anosmia
	A-6 (45, F)	7	moderate grade I	ageusia	anosmia
	A-7 (51, M)	5	moderate grade I	hyper-sensation for salty	anosmia
	A-8 (24, M)	5	mild	no complaint	anosmia
	A-9 (52, F)	5	moderate grade I	no complaint	anosmia
	A-10 (52, F)	5	mild	hyper-sensation for salty	anosmia
	A-11 (59, F)	6	mild	hypogeusia	no complaint
	A-12 (43, F)	3	mild	hyper-sensation for bitter	no complaint
	A-13 (64, M)	0	moderate grade II	hypogeusia	hyposmia
	A-14 (70, F)	3	mild	hypogeusia	anosmia
	A-15 (64, F)	5	mild	hyper-sensation for salty	hyposmia
	A-16 (60, M)	2	mild	hypogeusia	hyposmia
	A-17 (65, M)	1	mild	Ageusia	anosmia
	A-18 (48, M)	6	moderate grade I	hypogeusia	no complaint
	A-19 (75, F)	2	mild	hypogeusia	anosmia
	A-20 (74, F)	7	moderate grade I	hypogeusia	hyposmia
	A-21 (30, F)	1	mild	hyper-sensation for bitter	no complaint
	A-22 (67, M)	6	moderate grade II	hypergeusia	hyperosmia
	A-23 (40, F)	3	mild	ageusia	anosmia
	A-24 (61, F)	10	moderate grade I	ageusia	anosmia
	A-25 (47, F)	9	mild	hyper-sensation for salty/bitter	anosmia
	A-26 (35, F)	2	mild	hyper-sensation for salty/sour	no complaint

Group	Patient ID (Age, Sex)	pdo (days)	Severity of COVID-19	Dysgeusia	Dysosmia
**B**	B-1 (25, M)	5	moderate grade I	no complaint	no complaint
	B-2 (70, M)	0	mild	no complaint	no complaint
	B-3 (23, M)	3	mild	no complaint	no complaint
	B-4 (51, M)	4	mild	no complaint	no complaint
	B-5 (50, F)	4	mild	no complaint	no complaint
	B-6 (44, F)	3	mild	no complaint	no complaint
	B-7 (57, M)	4	moderate grade I	no complaint	no complaint
	B-8 (66, F)	7	moderate grade II	no complaint	no complaint
	B-9 (69, M)	5	moderate grade I	no complaint	no complaint
	B-10 (31, F)	3	mild	no complaint	no complaint
	B-11 (32, M)	2	moderate grade I	no complaint	no complaint
	B-12 (45, F)	7	moderate grade I	no complaint	no complaint
	B-13 (74, F)	1	mild	no complaint	no complaint
	B-14 (75, M)	4	moderate grade II	no complaint	no complaint
	B-15 (23, F)	4	mild	no complaint	no complaint
	B-16 (58, M)	3	mild	no complaint	no complaint
	B-17 (60, M)	1	mild	no complaint	no complaint
	B-18 (61, M)	1	mild	no complaint	no complaint
	B-19 (77, F)	5	moderate grade II	no complaint	no complaint
	B-20 (69, M)	5	moderate grade II	no complaint	no complaint
	B-21 (37, F)	2	mild	no complaint	no complaint
	B-22 (47, F)	1	mild	no complaint	no complaint
	B-23 (80, M)	9	moderate grade I	no complaint	no complaint
	B-24 (52, M)	6	mild	no complaint	no complaint
	B-25 (23, F)	5	mild	no complaint	no complaint
	B-26 (49, M)	0	moderate grade I	no complaint	no complaint

**Table 2:** Comparison of baseline factors and Trp metabolite concentrations in patients with COVID-19 with and without dysosmia/dysgeusia.

	**Group A**		**Group B**		***P***

**Mean Age**	52.77		51.85		0.33
**Sex**	M:F = 10:16		M:F = 15:11		0.38
**Mean pdo**	4.69		3.62		0.11
**Severity of COVID-19**	mild:moderate I:moderate II = 14:9:3		mild:moderate I:moderate II = 15:7:4		1
**Vaccination**	unvaccinated:vaccinated:unknown = 9:16:1		unvaccinated:vaccinated:unknown = 2:23:1		0.04

**Trp metabolites**	**Mean ± SD**	**Median (range)**	**Mean ± SD**	**Median (range)**	**Mann-Whitney *P***

5-HT (ng/mL)	90.91 ± 68.26	78.08 (8.17–106.45)	79.03 ± 34.69	85.58 (11.47–166.56)	0.73
Trp (μg/mL)	9.47 ± 2.18	9.70 (4.59–13.89)	11.68 ± 3.54	10.40 (7.52–13.34)	0.030
Kyn (ng/mL)	685.62 ± 308.36	599.36 (239.6–1763.5)	601.41 ± 175.62	576.70 (336.0–1164.4)	0.43
KTR ( × 10^3^)	79.25 ± 65.55	61.34 (40.47–384.2)	54.19 ± 16.95	53.52 (26.13–86.64)	0.037
KYNA (nM)	95.59 ± 100.30	74.76 (11.80–564.97)	95.52 ± 35.70	85.03 (47.65–109.03)	0.15
QUIN (nM)	1018.65 ± 2234.00	574.39 (100.39–11909)	443.65 ± 238.69	443.65 (83.09–998.3)	0.044

Patients with COVID-19 with (group A)/without (group B) dysosmia and/or dysgeusia are presented, respectively. Each group comprised 26 patients, with data collected including age, sex, post-day from the onset (pdo; the average timepoint at blood sampling after COVID-19 onset), and severity of COVID-19. Age, pdo, and disease severity were manually matched between groups. The mean age and pdo are summarized in Table 2, with no significant differences detected using the Mann–Whitney *U* test. Fisher's exact test was applied for intergroup comparisons of COVID-19 severity.

The mean ± SD and median (range; minimum-maximum) serum concentrations of Trp metabolites (5-HT, Kyn, Trp, KYNA, QUIN, and KTR) in patients with COVID-19 with (group A)/without (group B) dysosmia and/or dysgeusia are shown. For statistical analyses, the *t*-test was applied to age, sex, pdo, and disease severity. Fisher's exact test was used to compare vaccination status after excluding two patients with unknown status. The Mann–Whitney *U* test was applied to serum concentrations of Trp metabolites as continuous variables. *P* < 0.05 was considered statistically significant. M, mild; G I, moderate grade I; G II, moderate grade II; UV, unvaccinated: V, vaccinated; 5-HT, 5-hydroxytryptamine (serotonin); KTR, Kny/Trp ratio; Kyn, kynurenine; KYNA, kynurenic acid; QUIN, quinolinic acid; Trp, tryptophan, SD, standard deviation.

Summary of mRNA vaccination status of the enrolled participants with acute COVID in groups A and B and incidence of dysosmia/dysgeusia are presented. Dysosmia was correlated with a lack of mRNA vaccination. Two patients with unknown vaccination status were excluded from the analysis, as presented in Table 3. For statistical analyses, the *t*-test was applied to age, and Fisher's exact test was applied to sex, vaccination status, and symptom positivity. *P* < 0.05 was considered statistically significant.

**Table 3:** Prevalence of dysosmia/dysgeusia according to vaccination status.

	**Unvaccinated (n=11)**		**Vaccinated (n=39)**		**Chi-square *p*-value**

Mean age	48.82		52.77		0.33
Sex	M:F = 3:8		M:F = 11:18		0.49

	**Positive**	**Negative**	**Positive**	**Negative**	**Fisher's P value**

**Dysosmia**	8	3	12	27	0.012
**Dysgeusia**	6	5	15	24	0.340

The study period was from September 13, 2021 to September 30, 2023, thereafter, data was collected and accumulated intermittently between October 1, 2023, and June 30, 2024; we did not have access to information that identify individual participants after the data collection.

The inclusion criteria required that participants had documented taste/olfaction status during admission and an available blood sample. Patients were excluded if they could not be interviewed due to severe respiratory failure, cancer progression, or cognitive impairment. Adolescents aged <20 years, pregnant women, and individuals who did not comprehend Japanese were also excluded to prevent miscommunication. Other exclusion criteria included current smoking status, pre-existing taste or smell impairment, recent anti-cancer chemotherapy within three weeks before infection, and untreated or incompletely treated brain metastases or CNS invasion in patients with cancer or hematopoietic tumors (Fig. 1a).

### 2.2 Diagnosis and severity classification of COVID-19

SARS-CoV-2 RNA was detected using a nucleic acid test or antigen quantitative test from nasopharyngeal or pharyngeal swab samples obtained upon admission. In Japan, COVID-19 severity is classified into four categories based on the Japanese Ministry of Health, Labour and Welfare guidelines (Hara et al., 2022). Findings from high-resolution computed tomography (HRCT) of the chest were independently assessed by a pulmonologist (JT or SM) and a radiologist to determine disease severity. HRCT abnormalities, including ground-glass opacity, consolidation, and reticular fibrosis such as honeycombing, were considered indicative of COVID-19-associated pneumonia (Hara et al., 2022).

Mild cases were defined as those with slight symptoms and no pneumonia on HRCT. Moderate grade I cases exhibited mild respiratory symptoms with pneumonia (93 % < peripheral oxygen saturation [SpO2] <96 %) but did not require oxygen supplementation. Moderate grade II cases were those with respiratory failure (SpO2 ≤93 %) and pneumonia requiring oxygen supplementation. Severe cases included patients who required mechanical ventilation or extracorporeal membrane oxygenation support.

### 2.3 Evaluation of dysosmia and dysgeusia

A standardized assessment of patients' smell and taste sensations was conducted during hospital admission through interviews about the presence/absence of symptoms, using a structured checklist administered by a physician specializing in COVID-19 (JT) or duty nurses. Taste sensation was evaluated after the consumption of hospital-provided food, with specific categories for sweet (e.g., jam or sugar), salty (e.g., salt), sour (e.g., lemon or umeboshi [Japanese apricot]), bitter (e.g., Japanese tea or coffee), and umami (e.g., miso soup). Smell sensation was assessed by asking patients whether they could perceive scents, such as coffee, curry, fruits, or their own excrement.

### 2.4 Case-control study design

Regarding patients with pre-existing cancer, their original cancer diagnosis and treatment departments were resumed upon admission. In contrast, for other patients with COVID-19, physicians were assigned via a rotational system covering all clinical departments. All patients were admitted to a dedicated infection-specific ward, where a physician specializing in COVID-19 (JT) managed all COVID-19-related issues.

Of the 520 patients initially hospitalized with COVID-19 who underwent HRCT, 17 were excluded due to severe respiratory failure, cancer progression, or cognitive impairment that prevented interviews. Adolescents aged <20 years, pregnant women, and non-Japanese-speaking patients were also excluded to prevent miscommunication. Patients with available blood samples at admission and documented taste/olfaction assessments during hospitalization were selected as potential baseline participants, resulting in an initial cohort of 138 patients. From this cohort, patients who had received anti-cancer chemotherapy within three weeks before infection, those with untreated or incompletely treated brain metastases or invasive hematopoietic tumors in the CNS, those with pre-existing taste or smell impairment due to cancer or cancer therapy, and current smokers were excluded. A final cohort of 95 patients was selected (Fig. 1a). Among these, 26 patients with dysosmia and/or dysgeusia were classified as group A. The remaining 69 patients did not report dysosmia or dysgeusia; however, the distribution of COVID-19 severity [mild (M): moderate grade I (GI): moderate grade II (GII) = 51:9:9] and proportion of patients with cancer (23/69 = 33.33 %) differed substantially from those in group A [M: GI: GII = 14:9:3] and (14/26 patients = 53.85 %). Therefore, to construct a comparable control group, (group B), 26 patients were manually selected from this subgroup, matched to group A (without intergroup differences) for pdo, age, COVID-19 severity, and proportion of patients with cancer.

As primary analysis in this study, we examined the existence of intergroup differences in serum concentrations of KP metabolites. As secondary sub-analysis, we evaluated the association of mRNA vaccination with/without the emergence of these manifestations in both groups.

### 2.5 Blood sample collection

Blood samples were collected using a vacuum blood-collection vessel (Venoject II, Terumo, Tokyo, Japan) upon admission or at the first hospital visit. Serum was separated by overnight storage at 4 °C, followed by centrifugation at 2000×*g* for 10 min. The serum samples were stored at −80 °C until analysis.

### 2.6 Metabolites of the kynurenine pathway

Fig. 1b illustrates KP and Kyn formation via indoleamine 2,3-dioxygenase/Trp 2,3-dioxygenase (IDO/TDO), highlighting the role of glial cells in producing key metabolites. KYNA is predominantly synthesized by astrocytes, whereas QUIN is mainly produced by microglia and macrophages. QUIN also serves as a precursor for the de novo synthesis of nicotinamide adenine dinucleotide (NAD^+^), as depicted in Fig. 1b.

### 2.7 Measurement of serum metabolites

Serum metabolite concentrations were measured using enzyme-linked immunosorbent assay (ELISA) following the manufacturer's instructions. If necessary, minor modifications such as adjustments to incubation time and plate shaking were applied to standardize reactivity across different ELISA kit lots. Metabolites were quantified using competitive ELISA, which enabled precise detection of small molecules in samples with antiserum against each metabolite. The following ELISA kits were used: Serotonin ELISA (KA1894, Abnova, Taoyuan City, Taiwan), Tryptophan ELISA, Kynurenine ELISA, Kynurenic Acid ELISA, and Quinolinic Acid ELISA (BA-E−2700R, BA-E−2200R, IS-I-0200R, IS-I-0100R, respectively; ImmuSmol SAS, Bordeaux, France). Data were analyzed using the EnSpire instrument (PerkinElmer, Hamburg, Germany).

Additionally, the following laboratory parameters were measured: white blood cell count (reference interval [RI]: 3300–8600/μL), platelet count (RI: 15.8 × 10^4^−34.8 × 10^4^/μL), total bilirubin (T-bil; RI: 0.4–1.5 mg/dL), albumin (RI: 4.1–5.1 g/dL), creatinine (RI: 0.65–1.07 mg/dL [male], 0.46–0.79 mg/dL [female]), lactate dehydrogenase (RI: 124–222 U/L), C-reactive protein (RI: 0.000–0.140 mg/dL), Krebs von den Lungen-6 (RI: <500 U/mL), ferritin (RI: 39.4–340 ng/mL [male], 3.6–114 ng/mL [female]), zinc (RI: 80–130 μg/dL), and copper (RI: 68–128 μg/dL).

### 2.8 Statistical analysis

Statistical analyses were performed using EZR version 4.20 (Kanda, 2013). The chi-square test and Mann–Whitney *U* test were applied for categorical variables, such as the presence or absence of dysosmia/dysgeusia and vaccination status, as well as continuous variables, such as time-related variables and metabolite concentrations. The Shapiro–Wilk test was used to determine whether data followed a normal distribution, and the Mann–Whitney *U* test was applied for non-normally distributed data. A *p* value < 0.05 was considered statistically significant. Fisher's exact test was applied for intergroup comparisons of COVID-19 severity.

### 2.9 Study approval

This study was conducted in accordance with the Declaration of Helsinki and was approved by the Institutional Review Board of Kanagawa Cancer Center (approval no.: 2021eki-75). The privacy rights of all participants have been observed, and comprehensive consent for research conducted at the hospital was obtained from all patients before receiving their treatments. Informed consent for the present study was obtained through an opt-out option available on the hospital website (https://kcch.kanagawa-pho.jp/general/cr-kansenshou.html). The requirement for written informed consent was waived due to the quarantine mandates for patients with COVID-19, which prevented in-person consent during the COVID-19 pandemic, and also due to the retrospective nature of the study.

## 3 Results

### 3.1 Patient characteristics

Among the 520 patients with COVID-19 admitted to our hospital between September 13, 2021 and September 30, 2023, all underwent chest HRCT examinations as part of a case-control study. Following the application of initial inclusion and exclusion criteria, 95 patients were selected as potential participants for the baseline study (Fig. 1a).

A total of 52 patients were included in the final analysis and were stratified based on the presence (group A: n = 26) or absence (group B: n = 26) of dysosmia and/or dysgeusia (Table 1). No significant differences were noted in the average time of blood sampling after COVID-19 onset (post-day from onset, pdo) [4.69 ± 2.56 days in group A *vs*. 3.62 ± 2.26 in group B, *p* = 0.104] and age [mean ± SD: 52.77 ± 12.96 years in group A *vs*. 51.85 ± 18.11 years in group B, *p* = 0.934], using the Mann–Whitney *U* test. Additionally, no significant intergroup differences were found in intergroup comparisons of COVID-19 severity using Fisher's exact test.

### 3.2 Serum levels of 5-HT, Trp, and KP metabolites in patients with COVID-19

**Fig. 2:**
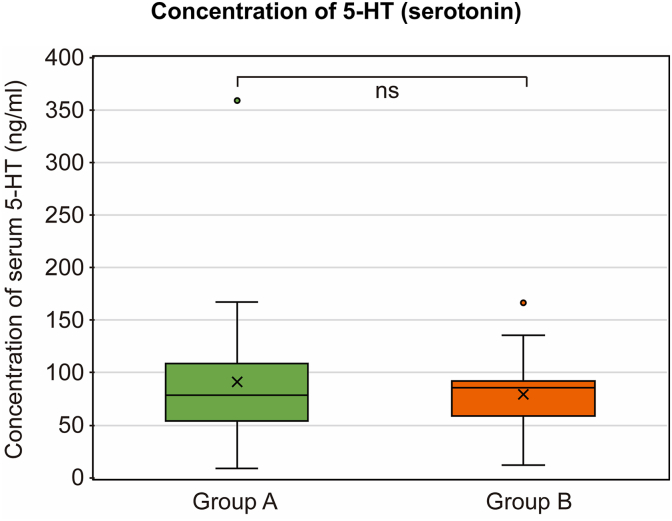
Concentration of serum serotonin (5-HT) in patients with COVID-19 with dysosmia/dysgeusia and without dysosmia/dysgeusia. There were no differences in serum 5-HT among (a) patients with COVID-19 and dysosmia/dysgeusia and (b) patients without dysosmia/dysgeusia. The Mann–Whitney *U* test was applied for intergroup comparisons. Each box plot represents the interquartile range (IQR) of the data, with the horizontal line inside the box indicating the median value. The lower and upper bounds of the box represent the first and third quartiles, respectively. The whiskers extend to the minimum and maximum values within 1.5 times the IQR from the lower and upper quartiles, respectively. The box plots display the distribution of values in serum concentration of 5-HT. The X- and Y-axes indicate each enrolled group (X-axis) and the concentration of serum 5-HT (Y-axis; ng/mL), respectively. COVID-19, coronavirus disease 2019; ns, not significant.

Representative data on Trp metabolites and clinical parameters are presented. in Table 2 and Supplementary Tables S1 and S2. Given its role as a key neurotransmitter for chemoreceptors and serotonergic neurons in the CNS, 5-HT concentration was measured. No significant difference was observed between group A [median: 78.08 ng/mL (range: 8.17–106.45)] and group B [median: 85.58 ng/mL (range: 11.47–166.56); *p* = 0.73] (Fig. 2).

**Fig. 3:**
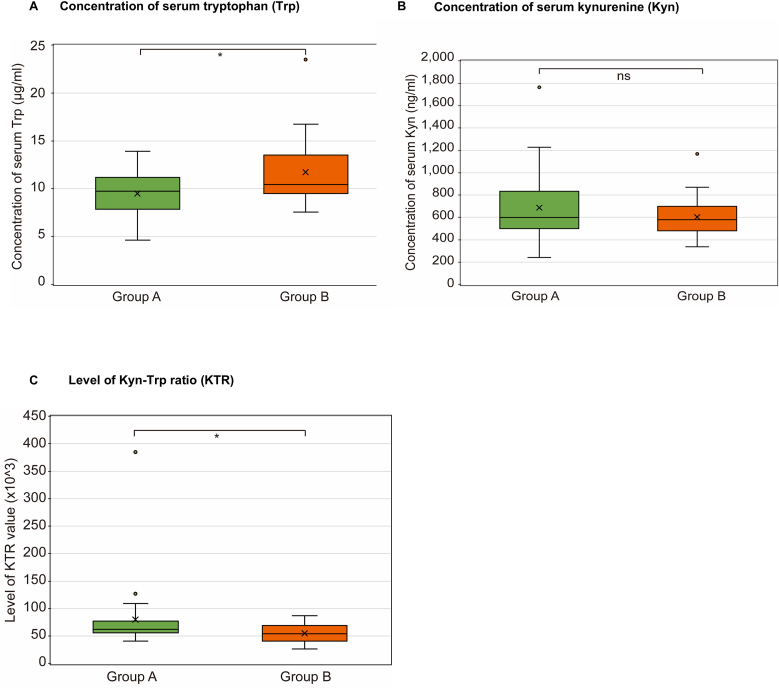
Concentration of serum tryptophan (Trp) and kynurenine (Kyn), and Kyn–Trp ratio (KTR) in patients with COVID-19 with and without dysosmia/dysgeusia. The Mann–Whitney *U* test was applied for intergroup comparisons. (a) Trp levels significantly decreased in patients with COVID-19 and dysosmia/dysgeusia (group A) compared with those without dysosmia/dysgeusia in group B (*p* < 0.031). (b) There were no significant differences in the Kyn levels between groups A and B. (c) The value of KTR is defined as Kyn/Trp × 10^3^. KTR was significantly elevated in group A compared with group B (*p* < 0.037). The box plots represent the median, lower, and upper quartiles, and whiskers correspond to the 1.5 × interquartile range. ns, not significant; ∗*p* < 0.05. The X- and Y-axes indicate each enrolled group (X-axis) and the concentration of serum (a) Trp (Y-axis; μg/mL), (b) Kyn (ng/mL), and (c) the value of KTR ( × 10^3^), respectively. COVID-19, coronavirus disease 2019.

**Fig. 4:**
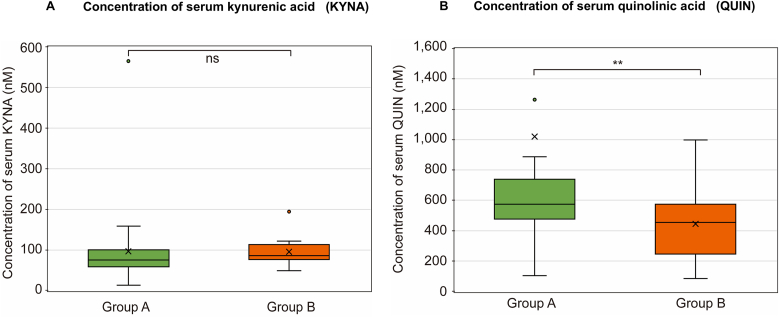
Concentration of Serum KYNA and QUIN in Patients with COVID-19 with and without Dysosmia/Dysgeusia. The Mann–Whitney *U* test was applied for intergroup comparisons. (a) There were no significant differences in the KYNA levels between Groups A and B. (b) QUIN levels in group A were elevated significantly compared to that in group B (*p* < 0.0169). The box plots represent the median, lower, and upper quartiles, and whiskers correspond to the 1.5 × interquartile range. ns, not significant; ∗∗*p* < 0.02. The X- and Y-axes indicate each enrolled group (X-axis) and the concentration of serum (a) KYNA (Y-axis; nM) and (b) QUIN (nM), respectively. COVID-19, coronavirus disease 2019; KYNA, kynurenic acid; QUIN, quinolinic acid.

Box plots illustrating the distribution of 5-HT and other Trp metabolites are presented in Fig. 2, Fig. 3, Fig. 4. Notably, no significant intergroup differences were observed in Kyn levels, serum Trp levels were slightly lower in group A [median: 9.70 μg/mL (range: 4.59–13.89)] than in group B [median: 10.40 μg/mL (range: 7.52–13.34); *p* = 0.031] (Fig. 3a and b). Further, KTR was significantly higher in group A [median: 61.34 × 10^3^ (range: 40.47–384.2)] than in group B [median: 53.52 × 10^3^ (range: 26.13–86.64); *p* = 0.037].

### 3.3 Relationship between the serum Kyn-to-Trp ratio (KTR) and dysosmia/dysgeusia

The KTR was calculated as serum Kyn/serum Trp × 10^3^. KTR was significantly higher in group A [median: 61.34 (range: 40.47–384.2)] compared to group B [median: 53.52 (range: 26.13–86.64); *p* = 0.037] (Fig. 3c).

### 3.4 Serum levels of downstream KP metabolites: KYNA and QUIN

For downstream metabolites in the KP pathway, KYNA levels were not significantly different between group A [median: 74.76 nM (range: 11.80–564.97)] and group B [median: 85.03 nM (range: 47.65–109.03); *p* = 0.15] (Fig. 4a). However, the QUIN levels were significantly elevated in group A [median: 574.39 nM (range: 100.39–11,909)] compared to group B [median: 443.65 nM (range: 83.09–998.3); *p* = 0.0169] (Fig. 4b).

### 3.5 Other clinical parameters and sub-analysis

Dysosmia was significantly associated with a lack of prior mRNA vaccination (*p* = 0.011; Table 3 and Supplementary Table S2). No significant associations were observed between dysosmia/dysgeusia and other clinical parameters. Additionally, disease severity did not differ significantly between the two groups (*p* = 0.859).

## 4 Discussion

This study presents several key findings. First, no significant differences were observed in 5-HT levels between the patients with COVID-19 with and without dysgeusia/dysosmia. Second, the Trp levels were significantly lower in patients with dysgeusia/dysosmia, while the Kyn levels showed no significant difference. However, the KTR was significantly higher in the dysgeusia/dysosmia group. Third, the QUIN levels were significantly elevated in patients with dysgeusia/dysosmia, whereas the KYNA levels remained unchanged. Fourth, dysosmia was strongly associated with anosmia or hyposmia and correlated with a lack of mRNA vaccination. In contrast, dysgeusia presented with a heterogeneous range of symptoms—mainly ageusia or hypogeusia, followed by hypersensitivity to salty taste—and showed no association with vaccination status.

We initially hypothesized that neurological symptoms, such as dysgeusia and dysosmia, in patients with COVID-19 might be linked to abnormal 5-HT modulation. This was based on the role of 5-HT as a shared neurotransmitter in chemoreceptors throughout the body and its direct innervation by serotonergic cranial nerves, such as the vagus nerve. Prior studies have reported decreased 5-HT levels in patients with COVID-19 (Saito et al., 2022; Thomas et al., 2020; Shen et al., 2020) and suggested that vagal dysfunction due to 5-HT depletion contributes to long COVID (Wong et al., 2023). However, our study found no significant differences in the 5-HT levels between the groups, suggesting that 5-HT dysregulation is not a primary factor in dysgeusia/dysosmia. Although a small amount of supplementary 5-HT is generated from Trp due to interception following KP activation, an insufficient time course for consumption of the stored 5-HT and the serum 5-HT level, which could be maintained at the same level in all the enrolled patients during acute COVID-19 (average timepoint at sample collection was <5 pdo), should be considered. 5-HT is a predictive biomarker of COVID-19 severity (Saito et al., 2022). Our participants comprised a population with qualities that did not lead to a considerable decrease in 5-HT levels, as their conditions were mild. Thus, 5-HT may be an irrelevant biomarker for dysosmia/dysgeusia, at least for acute COVID-19.

In addition to the 5-HT pathway, Trp is a substrate for the KP. More than 95 % of Trp in the human body is a substrate for the KP, generating several metabolites with distinct biological activities in immune- and neuromodulation (Vécsei et al., 2013; Stone et al., 2013). The major effects of COVID-19 are noted on Trp metabolism and the KP (Thomas et al., 2020). As an initial step of the KP, the conversion of Trp into Kyn is regulated by the rate-limiting enzyme IDO, which is induced by proinflammatory cytokines, such as interferon-γ, persistently upregulated in long-term COVID-19 (Krishna et al., 2024). IDO comprises two closely related family members: IDO1 and IDO2 (Wu et al., 2018). Although IDO2 is less active than IDO1, both mediate the metabolism of Trp to Kyn (Fatokun et al., 2013). Regarding their actions in the immune or nervous system, IDO induces immunosuppression through inhibition of effector T cells and differentiation of regulatory T cells. In pregnancy, the maternofetal tolerance in the placenta can be maintained (Munn et al., 1998). However, IDO leads to neurological defects via the production of neurotoxins, such as 3-hydroxykynurenine (3-HK), 3-hydroxyanthranilic acid (3-HAA), and QUIN (Krause et al., 2011; Stone et al., 2022). In addition to IDO, Kyn can be converted from Trp by another enzyme, TDO. The enzymatic activities of TDO and IDO are mainly maintained in the liver and extrahepatically, respectively (Vécsei et al., 2013; Stone et al., 2013; Schröcksnadel et al., 2006).

Given the lack of an assay for IDO, its activity is often measured by the KTR in blood in diseases characterized by excess inflammation, such as autoimmune and cardiovascular disorders and cancer (Schröcksnadel et al., 2006). As IDO1 is the most important contributor to IDO activity in the KP, the KTR is considered to have nearly equal activity as IDO1 (Wu et al., 2018; Opitz et al., 2020). However, considering the enhanced IDO2 activity in the lungs (Guo et al., 2022) and the presence of activated Kupffer cells (tissue-resident macrophages in the liver) (Detsika et al., 2022) in COVID-19, both IDO2 and TDO activities should not be considered negligible. In the present study, the KTR increased in patients with COVID-19 with dysgeusia/dysosmia than in those without dysgeusia/dysosmia. Thus, the emergence of dysgeusia/dysosmia could be related to the up-regulation of IDO/TDO activity.

Recently, Cysique et al. reported crucial findings on the etiology of long COVID wherein cognitive impairment was associated with anosmia and elevated Kyn level, KTR, and QUIN level because of long-lasting activated KP (Cysique et al., 2023). Based on the downstream metabolites of Kyn from two types of glial cells—KYNA from astrocytes and QUIN from microglia (Platten et al., 2019; Stone et al., 2022)—they estimated that an accumulation of QUIN released from SARS-CoV-2-infected microglia/macrophages is a potent contributor to long COVID. Our views on acute COVID-19 with dysosmia/dysgeusia aligned with theirs on long COVID. Owing to its activity as an agonist for N-methyl-d-aspartate receptors (NMDARs), QUIN exacerbates excitotoxic neuronal damage, whereas KYNA offers neuroprotective potential as an NMDAR antagonist (Vécsei et al., 2013; Stone et al., 2022). Although both QUIN and KYNA are prevented from bidirectional transmission because of the blood–brain barrier (BBB) in the steady state (Platten et al., 2019), elevated serum QUIN levels increase the permeability of the BBB (Torii et al., 2016). Furthermore, in addition to QUIN, studies have reported that increased interleukin-1β and tumor necrosis factor-α signaling can also increase the permeability of BBB and facilitate the CNS entry of viruses (Michael et al., 2016; Wang et al., 2004), and both cytokines are upregulated in COVID-19 (Huang et al., 2020). Namely, modulations of KYNA (which was unchangeable level in patients between with and without dysosmia/dysgeusia) and elevated QUIN level in the present study could reflect the activity of metabolite-producing cells (i.e., astrocytes and microglia/macrophages) in the CNS. Furthermore, increased QUIN level may also affect the immune system in COVID-19 patients with dysosmia/dysgeusia, as QUIN is a proinflammatory metabolite of KP, which has been shown to suppress Th1 responses through selective apoptosis (Badawy, 2023; Fallarino et al., 2020) and is also closely associated with organ fibrosis (Lahdou et al., 2013). Taken together, the imbalance in QUIN production from prolonged activated microglia/macrophages and KYNA from insufficiently activated astrocytes in COVID-19, which enhances neurotoxicity, may lead to the emergence of neurological manifestations such as dysosmia/dysgeusia or brain fog.

An outlier in QUIN levels was detected in one case (Patient ID: A-11) in the present study. This patient experienced prolonged renal dysfunction following anti-cancer therapy at the time of sampling, which contributed to a substantial standard deviation (SD) variation. However, the significant difference (*p* < 0.028) was maintained even after excluding the outlier, resulting in an improvement in SD. The physiological relationship between the QUIN levels and renal function, however, remains to be elucidated.

Taken together, the accumulation of QUIN from microglia/macrophages following production induced by activated IDO/TDO (high value of KTR) in the KP may be a consecutive shared mechanism in acute COVID-19 and long COVID. Further, secondary 5-HT depletion in long COVID (Wong et al., 2023) may lead to additional intricate malfunctions in the serotonergic CNS and chemoreceptors.

A close relationship between prolonged high QUIN level, dysosmia, and cognitive impairment in patients with long COVID has been reported (Cysique et al., 2023). Although the small sample size in our secondary sub-analysis limits the ability to definitive conclusions, a trend suggesting that mRNA vaccination may have a preventive effect on dysosmia, but not on dysgeusia, in acute COVID. The mRNA vaccine has effects on a broad range of cell-mediated immunity along with a specific-antibody production (Kakugawa et al., 2024; Kometani et al., 2024). In the present study, the observed preventive effects of mRNA vaccination on dysosmia, even if partial, may reflect a stabilizing effect of the excessive activated KP on SARS-CoV-2-infected cells. Furthermore, since different protective effects on dysosmia/dysgeusia were shown, the finding may indicate that dysosmia/dysgeusia were distinctively regulated and that the influence of the vaccine against dysosmia may militate stronger than that against dysgeusia by less activity of the KP in macrophage/glia cells. Furthermore, regarding the diversity of manifestations, anosmia and hyposmia accounted for the majority of dysosmic symptoms in the present study, whereas dysgeusia manifested heterogeneously, predominantly as ageusia or hypogeusia, followed by hypersensitivity to salty taste. In TB, different sensory neurons are specialized to detect specific compounds: sweet, bitter, and umami tastes are mediated by G-protein-coupled receptors, similar to olfactory receptors, while salty and sour tastes are sensed by ion-channel receptors (Barretto et al., 2015). The diversity of manifestations in dysgeusia may indicate different neurological abnormalities based on the distinctive mechanisms of signal transmission, which remain to be investigated.

Nonetheless, we did not examine NAD^+^, a metabolite far downstream of QUIN in the KP in the present study, as shown in Fig. 1b. NAD^+^ has indispensable functions in immune/nervous systems, including its function as a switch for macrophages to shift their polarization toward M2 (Covarrubias et al., 2021). M2 macrophages are crucial for severe COVID-19 (Hara et al., 2022; Wendisch et al., 2021). Notably, recruited M2, which are driven from the circulating monocytes into the lungs, are key mediators of COVID-19-associated-ARDS-induced lung fibrosis (Wendisch et al., 2021). Additionally, NAD^+^ can behave as a neurotransmitter or an endogenous agonist of P2Y1 cell-surface purinergic receptors (Fontecha-Barriuso et al., 2021; Mutafova-Yambolieva et al., 2007), which are also expressed in the vagus nerve that innervates pulmonary NEB, thereby controlling ventilation and heart rate (Chang et al., 2015). Pausing or inhibiting the hypoxia-ventilation response accompanied by relative bradycardia can be driven through the stimulation of P2Y1 receptors. Furthermore, P2Y1 receptor signaling can induce platelet aggregation and lead to thrombosis (Ribes et al., 2023). Thus, it is plausible that robust P2Y1 stimulation on vagal afferent neurons and platelets by excessive QUIN-induced-NAD^+^, which is released from SARS-CoV-2-hijacked dysfunctional macrophages/microglia in the lungs and the vagal nervous system, can lead to the mysterious silent hypoxemia (Brouqui et al., 2021; Couzin-Frankel, 2020). In addition to NEB, P2Y1-expressing neurons innervate other chemoreceptors, such as EC and TB (Linan-Rico et al., 2016; Prescott et al., 2020). Thus, as previously reported that SARS-CoV-2 infection intensifies NAD^+^ synthesis (Thomas et al., 2020), the effects of NAD^+^ on these chemoreceptors are critical, and the elevated QUIN is estimated to be a potent contributor to the up-regulation of NAD^+^ in acute COVID-19, although there are two pathways along with the KP (the Preiss–Handler and the Salvage pathways) concerning the NAD^+^ synthesis (Covarrubias et al., 2021). Nevertheless, the roles of metabolites downstream of QUIN, such as NAD^+^, in COVID-19 warrant further investigation.

Our study has some limitations. First, the present study did not include the enough targeted sample size and might be underpowered to reflect whether the initial hypothesis that serum concentrations of neuromediators synthesized from Trp would swing significantly between the groups of patients with COVID-19 with/without neurological manifestations. Moreover, our trial was limited with respect to assessing the severe disease of COVID-19, as we excluded severe cases of dangerous hypoxemia to avoid patient overburden and prioritize rescuing them, notably in the era of highly pathogenic variants of SARS-CoV-2 prevalence, such as the alpha and delta strains. As a result, there was no choice to reduce the sample size less than estimated in advance. Thus, as a retrospective study conducted at a single institution, the sample size could not have been controllable. Second, because our hospital was an oncology specialist center, patients with cancer or those who received anti-cancer chemotherapy were included. The participants confirmed that they were free from dysgeusia/dysosmia before the SARS-CoV-2 infection. Furthermore, because IDO1 may be induced by an undesirable effect of either cancer itself (Opitz et al., 2020) or anti-cancer agents (Creelan et al., 2013), patients who received anti-cancer therapy within 3 weeks before the onset of COVID-19 were excluded. Third, the evaluation of dysosmia/dysgeusia lacked standardized, validated tools in the present study, therefore, expert neurologists are required to perform quantitative assessments of these manifestations in future studies. Thus, clinical and functional data from a multi-center prospective study focusing on a specific cohort, using a well-defined patient group, are necessary for confirming the usefulness of the markers for neurological manifestations of COVID-19 and their integration into multiparametric findings based on neuroscience. Fourth, as we could not perform cerebrospinal fluid examinations to address physiological limitations related to BBB permeability, we could only infer that serum QUIN/KYNA levels may reflect the activity of both CNS glial cells and peripheral macrophages/glia-like cells in chemoreceptors in brain and peripheral tissues. Therefore, our findings might limit the ability to draw definitive conclusions and not be sufficiently generalizable.

However, from a different perspective, accurate and objective history-taking in large-scale trials of dysosmia/dysgeusia may be unfeasible in acute COVID-19, particularly during the prevalence of highly pathogenic SARS-CoV-2 variants, such as the alpha and delta strains. This is attributed to challenging circumstances, including infection-prevention measures, complex clinical conditions, and the need for rapid responses to more critical disorders, such as dyspnea and arrhythmia. Thus, there may be an advantage in simply conducting one-on-one, direct, timely interviews with a skilled physician using consistent language, allowing clear differentiation of the presence or absence of obvious smell/taste impairment.

Finally, as QUIN has a broad spectrum of antiviral action, including against SARS-CoV-2 (Zhao et al., 2022), patients may be protected from various superinfections. Thus, elevated serum QUIN and activated KP may represent a double-edged sword in COVID-19. Based only on the present data, whether the role of activated KP in COVID-19 is harmful to patients could not be determined, nevertheless, the current study is the first trial to demonstrate the link between serum concentration of Trp metabolites and patients with acute COVID-19 stratified by the presence/absence of smell/taste impairment. Additionally, the findings proposed here may pave the way for further research on other neurotoxins/mediators in the KP, such as 3-HK, 3-HAA, and NAD^+^. These discoveries could contribute to clinical practice by providing a powerful tool for evaluating impairments and improvements in neurological manifestations in the near future. This would enable quick, simple, and efficient follow-up through blood examinations in severe cases of dysosmia, dysgeusia, and other neuropathies, ultimately helping identify these cases and improve their prognosis. Moreover, future studies may enhance the understanding and treatment of IDO/TDO-induced neuropathies, such as dysosmia/dysgeusia, not only in COVID-19 but also in conditions like pregnancy or after anti-cancer chemotherapy, if the pathogenesis is found to be based on shared mechanisms of the KP.

## 5 Conclusion

This study demonstrated that the emergence of dysgeusia/dysosmia during acute COVID-19 is closely associated with the serum value of KTR (reflecting IDO/TDO activity) and QUIN, but not 5-HT. KTR and QUIN might be suggested as candidate biomarkers of dysosmia/dysgeusia in COVID-19. Controlling excessively activated KP and QUIN-producing macrophages/microglia and insufficiently activated KYNA-producing astrocytes can be a critical therapeutic target for treating COVID-19-induced long-lasting smell/taste impairment in both acute illness and long-term COVID.

## CRediT authorship contribution statement

**Jun Tsukiji:** Writing – review & editing, Writing – original draft, Visualization, Validation, Supervision, Resources, Project administration, Methodology, Investigation, Funding acquisition, Data curation, Conceptualization. **Shiro Koizume:** Validation, Methodology, Investigation. **Tomoko Takahashi:** Validation, Methodology, Investigation. **Shuji Murakami:** Validation, Investigation. **Hiroyuki Takahashi:** Validation, Software, Investigation, Formal analysis. **Sachiyo Mitsunaga:** Visualization, Validation, Investigation, Data curation. **Sho Nakamura:** Validation, Resources. **Hiroto Narimatsu:** Validation, Resources. **Yohei Miyagi:** Writing – review & editing, Validation, Supervision, Project administration, Funding acquisition.

## Data statement

Data are available within the manuscript and its supplementary materials.

## Funding

This work was supported by the Kanagawa Prefecture Hospital Cancer Fund [grant number 2022CancerC358]. The funder had no role in the study design, collection, analysis or interpretation of data, writing of the report or decision to submit the article for publication.

## Declaration of competing interest

The authors declare the following financial interests/personal relationships which may be considered as potential competing interests: Jun Tsukiji reports receiving a scholarship from Shionogi Pharma (the Kanagawa Prefecture Hospital Cancer Fund [grant number 2022CancerC358]). The other authors declare no conflicts of interest.

## Data Availability

Data are available within the manuscript and its supplementary materials.

## Glossary

ARDS: acute respiratory distress syndrome
BBB: blood–brain barrier
CB: carotid body
CNS: central nerves system
COVID-19: coronavirus disease 2019
EC: Enterochromaffin cell
ELISA: enzyme-linked immunosorbent assay
3-HAA: 3-hydroxyanthranilic acid
3-HK: 3-hydroxykynurenine
5-HT: 5-hydroxytryptamine (serotonin)
HRCT: high-resolution computed tomography
IDO: indoleamine 2,3-dioxygenase
IQR: interquartile range
KL-6: Krebs von den Lungen-6
KP: kynurenine pathway
KTR: kynurenine/tryptophan ratio
Kyn: kynurenine
KYNA: kynurenic acid
NAD: nicotinamide adenine dinucleotide
NEB: (pulmonary) neuroepithelial body
NMDAR: N-methyl-d-aspartate receptor
OBG: olfactory bulb glomeruli
QUIN: quinolinic acid
RI: reference interval
SARS-CoV-2: severe acute respiratory syndrome coronavirus 2
SCC: solitary chemoreceptor cell
SD: standard deviation
TB: taste bud
TDO: tryptophan 2,3-dioxygenase
Treg: regulatory T cell
Trp: tryptophan
